# Absence of SARS-CoV-2 in Placental Tissue After Maternal COVID-19

**DOI:** 10.1001/jamanetworkopen.2026.8567

**Published:** 2026-04-22

**Authors:** Shelli F. Farhadian, Benjamin Orlinick, Kristin M. Milano, Dilgash Mekael, Harvey J. Kliman

**Affiliations:** 1 Department of Medicine, Section of Infectious Diseases, Yale School of Medicine, New Haven, Connecticut; 2 Department of Epidemiology of Microbial Diseases, Yale School of Public Health, New Haven, Connecticut; 3 Department of Obstetrics, Gynecology and Reproductive Sciences, Yale School of Medicine, New Haven, Connecticut; 4 Department of Dermatology, Yale School of Medicine, New Haven, Connecticut

## Abstract

This case-control study examines whether SARS-CoV-2 protein or RNA persists in placental tissue following maternal COVID-19 infection.

## Introduction

SARS-CoV-2 infection during pregnancy has been linked to placental inflammation and fetal demise.^1,2,3^ Acute COVID-19 placentitis—marked by perivillous fibrin, monocytic intervillositis, and trophoblast necrosis—has been associated with stillbirth.^4^ Whether SARS-CoV-2 persists in placental tissue after maternal recovery remains unknown. We investigated whether SARS-CoV-2 protein or ribonucleic acid (RNA) persists in placental tissue months after maternal COVID-19 infection.

## Methods

This case-control study was conducted and reported in accordance with the Strengthening the Reporting of Observational Studies in Epidemiology (STROBE) reporting guideline. Placental specimens were collected at delivery between October 2020 and December 2024. Placentas were grouped as: (1) pre–COVID-19 negative control, (2) acute COVID-19 placentitis, and (3) recovered COVID-19 cases, including 3 stillbirths and 4 healthy full-term deliveries. Histologic sections underwent hematoxylin and eosin staining and immunohistochemistry for SARS-CoV-2 spike S1 (research resource identifiers [RRID]: AB_3717371), nucleoprotein (RRID: AB_2827974), and CD68 (RRID: AB_2314148)—concomitantly with appropriate positive and negative controls—as previously described.^5^ RNA in situ hybridization used Advanced Cell Diagnostics 2.5 LS Probe-V-nCoV2019-S (No. 848568), 2.5 LS Negative Control (No. 312038), and Leica UBC Positive Control (No. RS7760) probes on the Leica BOND-III stainer. RNA in situ hybridization positivity required distinct intracellular punctate trophoblast staining exceeding that of negative controls. All recovered cases were evaluated in parallel with acute COVID-19 placentitis and the prepandemic control placenta. Placental tissue was obtained under a waiver of consent for loss cases by the Yale University institutional review board and with informed consent for healthy deliveries through the Yale University Reproductive Sciences Biobank. Data were analyzed from July 2024 to July 2025, and Excel for Mac version 16 (Microsoft) was used for data organization.

## Results

Clinical and demographic characteristics of pregnancies pre-COVID-19 and following maternal SARS-CoV-2 infection are summarized in the Table. A total of 12 placentas from 3 groups were examined: pre-COVID control (1 [8.3%]), acute COVID-19 placentitis (4 [33.3%]), and pregnancies following recovery from maternal SARS-CoV-2 infection (7 [58.3%]). In acute COVID-19 placentitis, diffuse staining for the SARS-CoV-2 nucleoprotein protein was evident throughout the syncytiotrophoblast layer (Figure, A). SARS-CoV-2 RNA was also readily detected. These cases showed the classic triad of trophoblast necrosis, monocytic intervillositis, and perivillous fibrin deposition.

**Table.** Clinical Characteristics of Pregnancies Pre–COVID-19 and During and Following Maternal SARS-CoV-2 Infection

Case	Month and year of COVID-19	GA (rounded wk)	Vaccinated before delivery	Mode of delivery	Days from positive COVID-19 test to delivery	Outcome	Nucleoprotein IHC	SARS-CoV-2 ISH
PC control case								
PC1	NA	40	NA	NSVD	NA	Healthy newborn	Negative	Negative
AC placentitis cases								
AC1	October 2020^a^	25	No	IOL, vaginal	4	Stillbirth	Positive	Positive
AC2	September 2021	24	No	IOL, vaginal	6	Stillbirth	Positive	Positive
AC3	January 2022	31	No	IOL, vaginal	9	Stillbirth	Positive	Positive
AC4	December 2024	27	Yes	CD	3	Neonatal loss	Positive	Positive
CR cases								
CR1	June 2022	28	Yes	IOL, vaginal	150	Stillbirth	Negative	Negative
CR2	May 2022^a^	34	Yes	IOL, vaginal	153	Stillbirth	Negative	Negative
CR3	December 2021	22	Yes	IOL, vaginal	40	Stillbirth	Negative	Negative
CR4	December 2022	39	Yes	IOL, vaginal	126	Healthy newborn	Negative	Negative
CR5	December 2022^a^	40	Yes	NSVD	114	Healthy newborn	Negative	Negative
CR6	August 2022	41	Yes	IOL, CD	212	Healthy newborn	Negative	Negative
CR7	September 2022	40	Yes	IOL, vaginal	132	Healthy newborn	Negative	Negative

Abbreviations: AC, acute COVID-19; CD, cesarean delivery; CR, COVID-19 recovered; GA, gestational age; IHC, immunohistochemistry; IOL, induction of labor; ISH, in situ hybridization; NA, not applicable; NSVD, normal spontaneous vaginal delivery; PC, pre–COVID-19 control.

^a^ These cases are included in the Figure as representative images.

**Figure.**
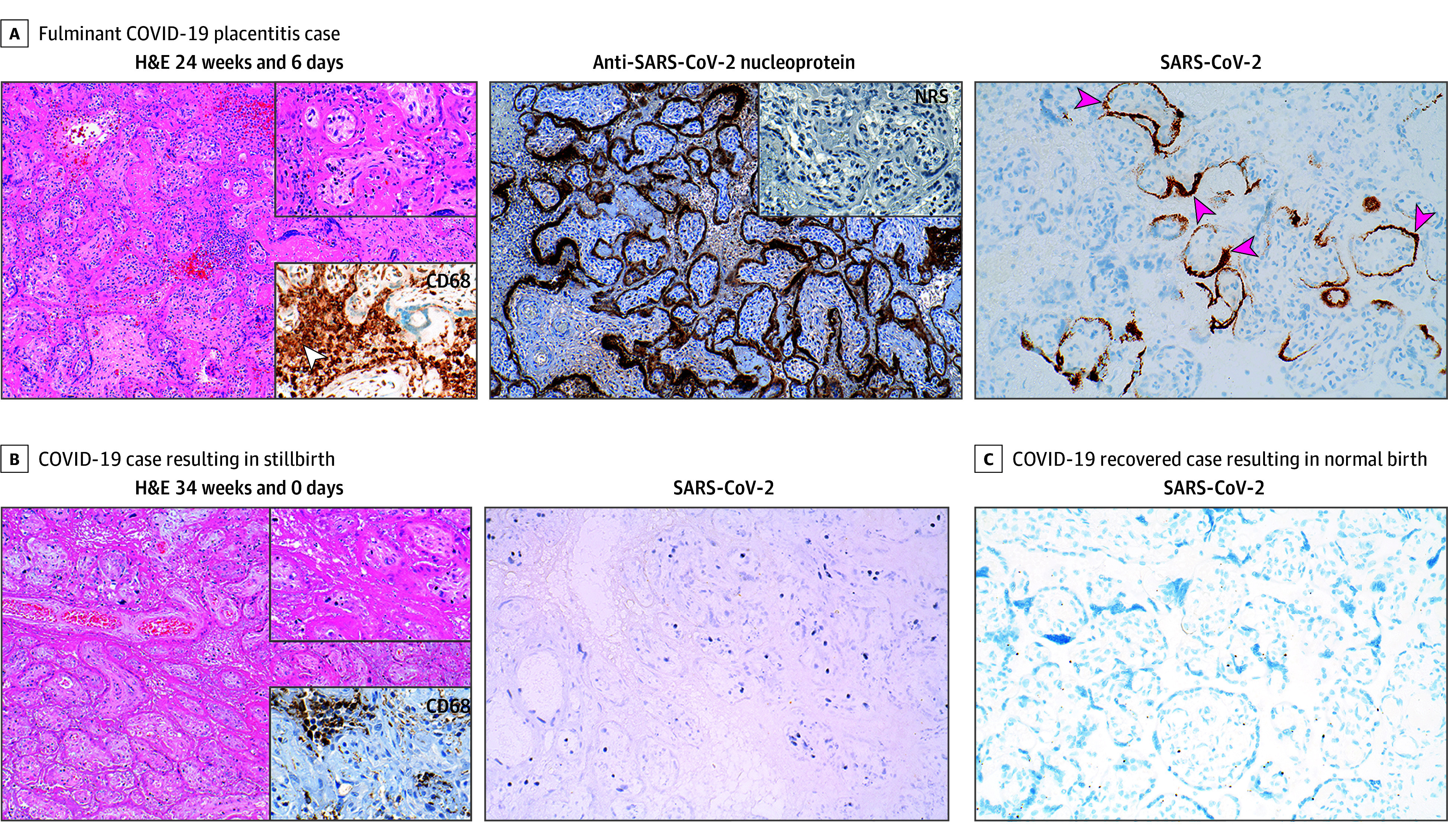
SARS-CoV-2 Nucleoprotein and RNA In Situ Hybridization in Acute COVID-19 Placentitis and After Maternal COVID-19 Recovery Panel A shows fulminant COVID-19 placentitis case delivered 4 days following a positive COVID test at 24 weeks and 6 days in an unvaccinated woman. Left: hematoxylin and eosin (H&E) staining and higher magnification H&E upper inset revealed classic intervillous fibrin deposition. CD68 lower inset identified marked numbers of intervillous monocytes (white arrowhead). Middle: Anti-SARS-CoV nucleoprotein immunostaining was intense, with diffuse syncytiotrophoblast staining. Normal rabbit serum (NRS) was negative (inset). Right: SARS-CoV-2 RNA probe stained about half of the syncytiotrophoblasts (pink arrowheads). Panel B shows a COVID-19 case resulting in stillbirth. Left: An H&E with a higher magnification H&E upper inset and CD68 lower inset of a COVID-19 recovered case resulting in a stillbirth at 34 weeks and 0 days at 153 days following a positive COVID-19 test. Right: SARS-CoV-2 RNA in situ staining in this same case, which was uniformly negative. Panel C shows uniformly negative SARS-CoV-2 RNA in situ staining in a COVID-19 recovered case resulting in a normal healthy liveborn at 39 weeks and 4 days where the patient had confirmed COVID-19 at 114 days prior to delivery. The panels shown are representative examples from each cohort; all cases were evaluated in parallel, and the results are summarized in the Table. All panels were photographed with a ×20 objective; all insets were further magnified by a factor of 2.

Among 7 placentas obtained from women who had recovered from SARS-CoV-2 infection, 3 resulted in stillbirth (42.9%) (Figure, B) and 4 resulted in healthy full-term live births (57.1%) (Figure, C), with placentas obtained 40 to 212 days after maternal infection. No evidence of viral persistence was identified. All cases were negative for nucleoprotein and SARS-CoV-2 RNA, even in the presence of residual inflammatory lesions.

## Discussion

This study demonstrates an absence of detectable SARS-CoV-2 protein or RNA in placental tissue obtained as early as 40 days after maternal infection, suggesting that persistent placental infection is unlikely following clinical recovery. While viral RNA or antigen has been reported in other tissues after acute infection, detection of viral components does not necessarily indicate a biologically active or replicating viral reservoir, and our findings do not support ongoing placental infection in the postacute period.

Among recovered cases resulting in a loss, placental injury was observed in the absence of detectable viral protein or RNA. These results build on prior studies of placental morphology by providing direct molecular evidence—through immunohistochemistry and RNA in situ hybridization—that SARS-CoV-2 protein and RNA are absent in placentas weeks to months after maternal infection, even in cases with persistent inflammatory changes.^6^ These findings suggest that postacute placental pathology may reflect immune-mediated, vascular, or reparative processes rather than ongoing viral infection.

This study is limited by its small sample size and retrospective design, which preclude precise estimation of the frequency of placental viral persistence and limit generalizability across populations, variants, and vaccination statuses. However, these findings indicate that SARS-CoV-2 protein and RNA are not detectable in placental tissue months after maternal COVID-19, arguing against placental viral persistence after recovery.

## Notes

Supplement.
Data Sharing Statement
