# Supplementary material for: Absence of SARS-CoV-2 in Placental Tissue After Maternal COVID-19
Source: JAMA Netw Open. 2026 Apr 22;9(4):e268567. doi: 10.1001/jamanetworkopen.2026.8567 (PMC13103815; doi:10.1001/jamanetworkopen.2026.8567)
Supplement: Supplement. — Data Sharing Statement [file jamanetwopen-e268567-s001.pdf]

Data Sharing Statement

Farhadian. Absence of SARS-CoV-2 in Placental Tissue After Maternal COVID-19. *JAMA Netw Open*. Published April 22, 2026. doi:10.1001/jamanetworkopen.2026.8567

Data
Data available: Yes
Data types: Deidentified participant data
How to access data: To obtain deidentified participant data, please email either harvey.kliman@yale.edu or shelli.farhadian@yale.edu.
When available: With publication

Supporting Documents
Document types: None

Additional Information
Who can access the data: No limitations.
Types of analyses: Any purpose.
Mechanisms of data availability: With investigator support.
Any additional restrictions: None.
